# A non-invasive soil-based setup to study tomato root volatiles released by healthy and infected roots

**DOI:** 10.1038/s41598-020-69468-z

**Published:** 2020-07-29

**Authors:** Sneha Gulati, Max-Bernhard Ballhausen, Purva Kulkarni, Rita Grosch, Paolina Garbeva

**Affiliations:** 1 Plant-Microbe Systems, Leibniz Institute of Vegetable and Ornamental Crops (IGZ) e.V., Theodor-Echtermeyer-Weg 1, 14979 Großbeeren, Germany; 2 Department of Microbial Ecology, Netherlands Institute of Ecology (NIOO-KNAW), Droevendaalsesteeg 10, 6708 PB Wageningen, The Netherlands; 3 Present Address: Institut für Biologie, Freie Universität Berlin, Altensteinstr. 6, 14195 Berlin, Germany; 4 Present Address: Translational Metabolic Laboratory, Radboud University Medical Center, Nijmegen, The Netherlands

**Keywords:** Microbiology, Plant sciences

## Abstract

The role of root exudates in mediating plant–microbe interactions has been well documented. However, the function of volatile organic compounds (VOCs) emitted by plant roots has only recently begun to attract attention. This newly recognized relevance of belowground VOCs has so far mostly been tested using systems limited to a two-compartment Petri-dish design. Furthermore, many of the plant–microbe interaction studies have only investigated the effects of microbial VOCs on plant growth. Here, we go two steps further. First we investigated the volatile profile of healthy and pathogen (*Fusarium oxysporum*) infected tomato roots grown in soil. We then used a unique soil-based olfactometer-choice assay to compare the migration pattern of four beneficial bacteria (*Bacillus* spp.) towards the roots of the tomato plants. We demonstrate that the blend of root-emitted VOCs differs between healthy and diseased plants. Our results show that VOCs are involved in attracting bacteria to plant roots.

## Introduction

The rhizosphere is an important niche housing complex interactions occurring between plant roots and (micro)organisms^1^. Belowground microbial interactions mostly happen via chemical communication^2^. Plants can release up to 20% of total photosynthetically fixed carbon in the form of root exudates, including volatile organic compounds (VOCs), into the rhizosphere, the interface between plant roots and the surrounding soil^3,4^. The chemical complexity of root exudates and their numerous roles in biotic belowground interactions has been well documented^5^. Plants are known to emit an enormous spectrum of VOCs; most of them are lipophilic, have a low molecular mass (< 300 Da) and a high vapor pressure (0.01 kPa or higher at 20 °C). This allows VOCs to migrate over long distances through the atmosphere. So far, more than 1,000 of these low molecular weight organic compounds are known, such as benzenoids, fatty acid derivatives, terpenoids, C5-branched compounds, various nitrogen, and sulfur containing compounds^6–8^. Some of these VOCs are constitutively emitted by healthy plants^9^ and recent experimental evidence suggests that they play an indispensable role in mediating long-distance interactions between organisms, such as recognition, attraction and defense^10,11^. Recent years have witnessed a surge in studies related to aboveground plant VOCs; however, only a few studies have focused on belowground volatile mediated interactions^2,9,12^. Some of the reasons for this are the underground nature of the roots and technical difficulties faced in designing realistic test systems for analyzing volatile-mediated interactions in soil.

Stress in plants can induce the emission of larger amounts of VOCs or even lead to synthesis of specific VOCs^13^. It has been broadly reviewed that plant exposure to biotic and abiotic factors affects the release of rhizodeposits into soil^4,14^. The chemical composition and intensity of induced plant VOCs can carry information about the physiological status of the plant, which could possibly serve as a cue for specific microbes. Much evidence exists to support the hypothesis that root-associated microbes (pathogenic or beneficial) alter the expression of belowground plant VOCs^15^. However, whether the volatile blend emitted by the plant upon pathogenic infection attracts or inhibits movement of bacteria to the roots has not yet been investigated.

In contrast to the limited knowledge about root volatile-mediated interactions, more studies have focused on the effect of microbial VOCs on plant growth or plant–plant interactions^5,16,17^. Many of these studies use two-compartment Petri dishes and artificial agar media^18,19^. However, the use of agar systems is questionable since it has been shown that the release of VOCs can greatly depend on the type of medium used^20^. Research that focuses on the impact of root produced VOCs on rhizosphere-associated bacteria is still rare^11,21^. Most of the conclusions are drawn from *Arabidopsis thaliana* plant model systems which provide insights into the role of VOCs produced by belowground plant parts; however extrapolation to agricultural or horticultural crop systems is controversial^11,22^. Therefore, studying the role of root-emitted VOCs in their interaction with the soil environment requires innovative tools that mimic real soil systems.

Our goal was to establish an experimental approach to improve our understanding of the role of root-emitted VOCs in the long-distance belowground interactions between the plant and applied beneficial bacteria. Most published studies focus on root VOCs produced by important crops such as *Zea mays*, *Citrus* spp*.* and *Brassica* spp. With this current study, we shed light on the root VOCs emitted by tomato (*Solanum lycopersicum*), which is one of the world’s most economically important vegetable crops. Tomatoes are grown in practically every country, in outdoor fields, greenhouses and nethouses, especially in Europe. Plants are often infected by soil-borne pathogens. Therefore, we investigated whether the infection of tomato plants with the fungal pathogen *Fusarium oxysporum* alters the root VOCs profile. In addition, we investigated whether the root-emitted VOCs attract bacteria to the root. For this purpose, we designed and applied a unique olfactometer-choice assay to assess the migration of applied beneficial bacteria towards the roots of tomato in a soil system. This olfactometer was used to test our hypotheses that (1) root VOCs can recruit bacteria from a distance, and (2) *F. oxysporum* infected tomato roots produce VOCs to attract the beneficial bacteria as a biocontrol measure against the pathogen.

## Material and methods

### Plant cultivation and pathogen inoculation

**Figure 1:**
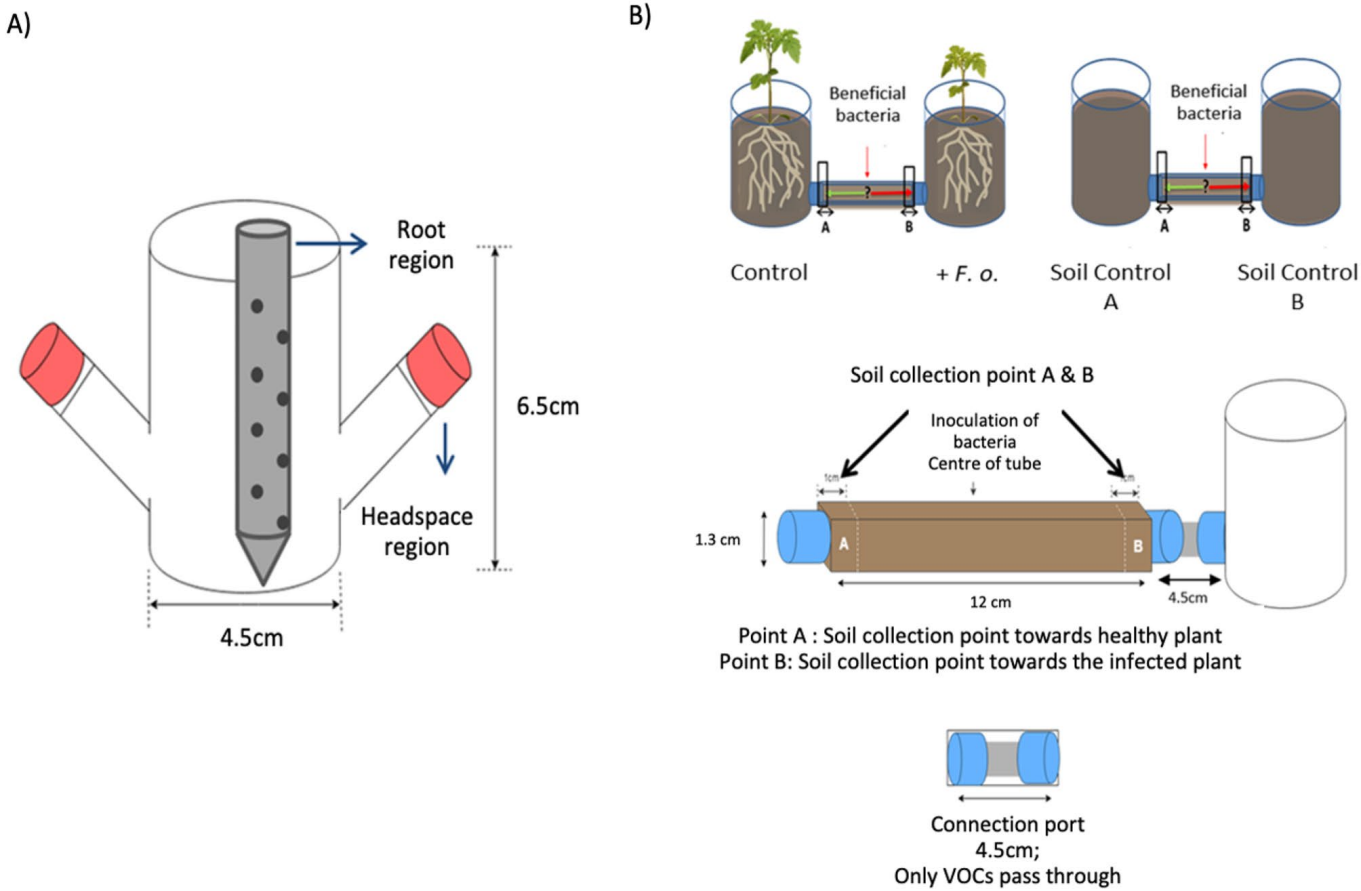
Glass assemblies used (**A**) to collect the root volatiles of tomato (cv. Hildares) released in the rhizosphere by the plant or by the pathogen *Fusarium oxysporum* (strain Fol007) in the soil. The metal trap with perforations (in grey) inserted in the soil was used to collect the volatiles from the root region, whereas the volatiles from the headspace region were collected from the end represented in orange (**B**) Olfactometer design used to assess the migration of bacteria towards the roots of healthy (healthy plant) and with *F. oxysporum* (infected plant) infected tomato plants.

Tomato seeds (*S. lycopersicum*) of cv. Hildares (Hild Samen GmbH, Marbach, Germany), susceptible to *F. oxysporum*, were surface sterilized with 0.8% sodium hypochlorite for 3 min followed by 70% ethanol for 1 min and rinsed six times with sterile distilled water. The seeds were incubated on half-strength Murashige and Skoog (MS) Medium (PlantMediaTM, United States) supplemented with agar (8 g L^−1^) for 3 days at 25 °C. Surface sterilized seeds were sown in the soil-filled part (gamma sterilized sandy soil) of the sterilized glass assemblies^23^ (Coelen Glastechniek, Cuijk, The Netherlands) and cultivated at 16 h/8 h at 25 °/20 °C day-night-cycle (400 µmol PAR m^−2^ s^−1^) (Fig. 1A). The plants were watered with sterile half-strength Hoagland solution (590.4 µg mL^−1^ Ca(NO_3_)_2_·4H_2_O, 253.0 µg mL^−1^ KNO_3_, 68.1 µg mL^−1^ KH_2_PO_4_, 246.5 µg mL^−1^ MgSO_4_·7H_2_O, 2.9 µg mL^−1^ H_3_BO_3_, 1.8 µg mL^−1^ MnCl_2_ 4H_2_O, 0.2 µg mL^−1^ ZnSO_4_·7H_2_O, 0.1 µg mL^−1^ CuSO_4_·5H_2_O, 0.1 µg mL^−1^ Na_2_MoO_4_·2H_2_O, and 41.5 µg mL^−1^ ferric EDTA). The soil moisture content was maintained at 13.4% w/w.

Tomato seedlings were inoculated with 2 mL of *F. oxysporum* conidia suspension (10^5^ mL^−1^) at the 1–2 leaf stage. Similarly, sterilized soil (without plants) was inoculated with *F. oxysporum* conidia suspension (10^5^ mL^−1^) and control plants were mock inoculated using sterile distilled water.

### Microbial inoculum preparation

The strain *F. oxysporum* Fol007 was grown on Potato dextrose agar (PDA, Merck, Darmstadt, Germany) and a micro-conidia suspension was obtained as described in a previous study^24^. Briefly, under sterile conditions, fungal culture plates were flooded with sterile water and the resulting conidia suspension was filtered through filter paper to separate the micro-conidia from the mycelium. The micro-conidia suspension was concentrated by centrifugation at 3,000×*g* for 10 min and adjusted to a density of 10^5^ micro-conidia mL^−1^. Infection of tomato roots by the pathogen was assessed using a specific primer pair (Forward primer: GAC GGT GTT TAT TCG GAT GG**;** Reverse primer: AGT TGC GCG ATA TGT GTT TG) for SIX1 gene amplification of *F. oxysporum.*

Four beneficial bacterial strains (F10: *Bacillus megaterium*; F15: *Bacillus licheniformis*; IOF49: *Bacillus* sp.; OF10: *Bacillus* sp.) were used as a consortium in this study. The strains were selected based on their plant growth promotion effect in vivo and their antifungal activity in vitro in a previous study (data not shown). Bacterial inoculum of the four bacterial strains were prepared in 10% Nutrient Broth medium (Carl Roth, Germany) overnight. Pure cell suspensions of the four bacterial strains were washed thrice with sterile phosphate buffer (10 mM KH2PO4, pH 6.5) and adjusted to a density of 10^6^ colony forming unit (CFU) mL^−1^.

### Volatile collection from healthy and pathogen infected tomato roots

The cultivation of tomato plants in a round glass assembly allows the collection of root-emitted VOCs from healthy plants and from with *F. oxysporum* infected plants (Fig. 1A). The root VOCs were collected after 7 days post inoculation at the 3–4 leaf stage.

The cylindrical glass vessel (4.5 cm diameter and 6.5 cm height) had two open outlets on the side which were closed with screw caps containing Polytetrafluoroethylene (PTFE) seals (Duran group GmbH, Wertheim/Mainz, Germany) until the collection of VOCs^11^. Metal holders with perforations were also inserted in the sterilized soil to collect the VOCs close to the roots. The metal holders were sealed with parafilm until collection of VOCs. For the collection of VOCs, steel traps filled with 150 mg Tenax TA and 150 mg Carbopack B (Markes International Ltd, Llantrisant, UK) were inserted in the metal holders and on one of the outlets. VOCs released into the soil were collected using steel traps filled with 150 mg Tenax TA and 150 mg Carbopack B (Markes International Ltd, Llantrisant, UK). VOCs were sampled from two locations in the glass vessel; one was collected by placing the steel traps in the metal holder with perforations enabling the passage of the root VOCs from the soil (root region) and the other from one of the two empty space outlets of the glass vessel (headspace region) (Fig. 1A). For each treatment, VOCs of four replicates (n = 4) were collected simultaneously by two traps per vessel. The steel traps were inserted and VOCs were passively absorbed for 24 h. Traps were removed, capped and stored at 4 °C until analysis using gaschromatography/quadrupole time-of-flight mass spectrometry (GC/Q-TOF).

### GC/Q-TOF analysis of the VOCs

The VOCs were desorbed from the Tenax traps using an automated thermodesorption unit (Unity TD-100 Markes International Ltd, Llantrisant, UK) with Helium flow at 50 mL min^−1^ at 210 °C for 12 min and were trapped on a cold trap at − 10 °C GC/Q-TOF (model Agilent 7890B GC and the Agilent 7200A QTOF, Santa Clara, CA, USA)^11,25,26^. The cold traps were heated at 280 °C for 3 min and introduced to the GC/Q-TOF (model Agilent 7890B GC and the Agilent 7200A QTOF, Santa Clara, CA, USA). A 30 mm × 0.25 mm ID RXI-5MS, film thickness 0.25 μm (Restek 13424-6850, Bellefonte, PA, USA) column was used, and the split ratio was fixed to 1:10. The following temperature program was used: 39 °C for 2 min, from 39 to 95 °C at 3.5 °C min^−1^, then to 165 °C at 6 °C min^−1^, to 250 °C at 15 °C min^−1^ and finally to 300 °C at 40 °C min^−1^, hold 20 min. VOCs were detected using the MS operating at 70 eV in EI mode. Mass spectra were acquired in full-scan-mode and compounds identified in a workflow as described before^11,25^. Briefly, MassHunter Qualitative Analysis Software V B.06.00 Build 6.0.633.0 (Agilent Technologies, Santa Clara, CA, USA) was used to extract the mass spectra which were converted to mzData files for further processing in MZmine V2.14.2. The files were imported to MZmine V2.14.2 (Copyright 2005–2012 MZmine Development Team;^27^). The VOCs were identified via their mass spectra using two libraries NIST 2014 V2.20 (National Institute of Standards and Technology, USA) and Wiley 7th edition spectral libraries and their linear retention indexes (LRIs). Calibration of LRI values was performed using an alkane calibration mix before compound identification using AMDIS 2.72 (National Institute of Standards and Technology, USA). The experimental LRI values were compared with the values in NIST and the in-house NIOO LRI database. After deconvolution and mass identification, peak lists containing the mass features of each treatment group were created and exported as CSV files for multivariate statistical analysis.

### Direct analysis in Real Time Mass Spectrometry of root metabolites

The metabolite profiles in the roots of healthy and infected plants were analyzed using Direct Analysis in Real Time Mass Spectrometry (DART-MS). This allows one to determine metabolites in a range of m/z 50–1,500. The DART mass spectrometry set-up comprises a DART ion source (model DART-SVP, IonSence, Saugus, USA) coupled with a Q Exactive Focus high-resolution mass spectrometer (Thermo Fisher Scientific, San Jose, CA, USA). The DART-MS was used to acquire the mass spectra in both negative and positive ion mode with the ion source parameters of 250 V (grid voltage) at 450 °C (gas heater temperature). Spectra were obtained over the mass range of m/z 50–1,500 at one spectrum per second. The helium flow rate for the DART source was 2.0 L s^−1^. Clippings of tomato root were sampled directly by gripping each sample longitudinally on a glass slide using double-sided tape and suspending it between the ion source and the mass spectrometer inlet. The slide was mounted on a linear rail system (IonSense, Saugus, MA, USA) that moved laterally from left to right through the open air space between the ion source and the mass spectrometer inlet at a rate of 0.8 mm s^−1^ as described in an earlier study^28^. Putative identification of the spectra acquired through DART-MS was performed by first subtracting the slide background spectra using X Caliber (Xcalibur 3.1). The peak alignment of the spectra was performed using MZmine. Compound identification of the spectra in positive and negative mode was performed using the KEGG database.

### Evaluation of bacteria attraction by root-emitted VOCs

A glass assembly combined with olfactometer was used to analyze the attraction of bacteria by VOCs emitted by non-infected and *F. oxysporum* infected tomato roots (Fig. 1B). The glass olfactometer (as described by Schulz-Bohm et al.^11^) was adapted and modified. Two glass vessels (4.5 cm diameter, 6.5 cm height) were connected to each other using one rectangular glass tube 14.0 cm in length (12 cm inner length) and 1.5 cm height (1.3 cm inner height) (Coelen Glastechniek, Cuijk, The Netherlands) (at a height of 1.5 cm from the bottom) via screw thread adapter couplings (4.0 cm length) with integral PTFE-faced silicone seals (DURAN Group GmbH, Wertheim/Main, Germany). The rectangular glass tubes were open at the top to enable filling with sterile soil and inoculating the bacteria. The glass tubes were closed with a sterile glass cap and covered with Parafilm.

The airspace between the glass vessel and the soil-filled part in the glass tube was about 4.5 cm. A nylon membrane mesh (Plastok Associates Ltd, Birkenhead, UK) (size:1 μm) was placed in between the glass vessel and screw thread adapters to prevent *F. oxysporum* hyphae passing from the soil into the glass vessels and connecting glass tubes. The tomato plants were cultivated and inoculated with the pathogen as described above. Seven days post pathogen inoculation, the design was assembled under sterile conditions. The glass tubes were filled with 10 g of sterile soil mixed with 0.8 mL of phosphate buffer. At the center of each glass tube 100 µL of bacterial suspension comprising four different bacterial strains (F10: *B. megaterium*; F15: *B. licheniformis*; IOF49: *Bacillus* sp.; OF10: *Bacillus* sp.) was inoculated. Four biological replicates (n = 4) of the olfactometer assembly were set up. For the control, soil-filled glass tubes were connected to both ends of the tube (n = 3). Based on the in vitro motility assays carried out for beneficial bacteria, a time frame of 72 h was chosen to assess the migration of beneficial bacteria (Supplementary Table S3). Soil was collected from both ends of the 12 cm olfactometer tube after 72 h (1 cm from the connection points at each end). The density of bacteria in the collected soil was quantified by qPCR and samples were stored at − 20 °C until DNA extraction.

### DNA extraction and qPCR for assessment of bacteria density

For assessment of bacterial attraction by VOCs emitted by tomato roots the density of all bacteria was quantified by qPCR as described before^11^. DNA was extracted from the soil using the manufacturer’s protocol for the DNAeasy PowerSoil Kit (Qiagen Benelux B.V., Venlo, The Netherlands), confirmed by NanoDrop and stored at − 20 °C until further use. All qPCR assays were performed with a BioRad CFX96 C1000 Touch Thermal cycler. 20 μL of a reaction mixture was used to amplify 16S rRNA genes of *Bacillus* sp. consisting of onefold SensiFAST SYBR No-ROX Kit (GC biotech B.V., Alphen aan den Rijn, The Netherlands), BSA (0.5 μg μL^−1^), 375 nM forward and reverse primers [BacF (5′-GGGAAACCGGGGCTAATACCGGAT-3′) and 1378R (5′-CGGTGTGTACAAGGCCCGGGAACG-3′)] and 5 μL of DNA (2–6 ng μL^−1^)^29^. DNase- and RNase-free water was used as no-template controls and was included in every qPCR run. The thermal cycling program was as follows: 5 min initial denaturation at 95 °C, followed by 40 cycles of denaturation for 30 s at 95 °C, annealing for 20 s at 55 °C, elongation for 20 s at 72 °C, and fluorescence signal detection for 15 s at 77 °C. Immediately after the 40th PCR cycle, melting curve analyses from 62 to 95 °C with increments of 1.0 °C was performed.

### Statistical analysis

Statistical analysis was performed using the program MetaboAnalyst V3.0, www.metaboanalyst.ca^30^. Data were normalized via log-transformation. One-way-ANOVA was combined with Tukey test (HSD-test) to identify significant abundant mass features (*p* ≤ 0.05). Important mass features in the samples were identified using PLS-DA analysis. Plant characteristics were analyzed by one-way-ANOVA combined with Tukey test (HSD-test) using IBM SPSS Statistics 23 (IBM, Somers, NY, USA).

## Results and discussion

### Plant growth

**Figure 2:**
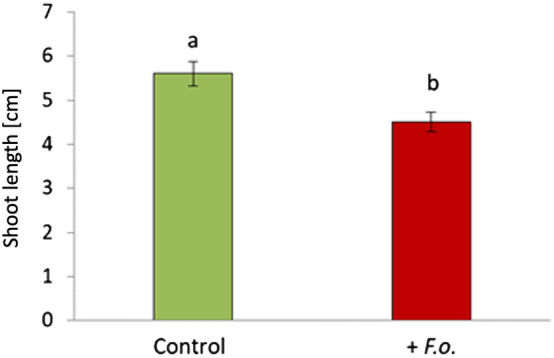
Shoot length of tomato plants (cv. Hildares) without pathogen (Control) and with inoculation of *Fusarium oxysporum* (strain Fol007) (+*F.o.*) 7 days after pathogen inoculation. Different letters on the top of error bars represent significant differences according to Tukey test (*p* ≤ 0.05).

The well-known soil-borne plant pathogen *F. oxysporum* is an ascomycetous fungus causing Fusarium wilt disease in tomato plants^31^. In this study, we assessed the impact of the pathogen *F. oxysporum* Fol007 on tomato growth by measuring shoot length 7 days after pathogen inoculation. Colonization of tomato roots by *F. oxysporum* Fol007 was confirmed by PCR assays using pathogen-specific primers (Supplementary Fig. S1). The shoot length of inoculated plants was significantly reduced compared to healthy plants (*p* ≤ 0.05) (Fig. 2). Reduction in the shoot length could be a consequence of the pathogen blocking the xylem vessels, leading to impaired plant growth^32^. Our results are consistent with other studies highlighting that infection of tomato plants with *F. oxysporum* leads to a significant reduction in shoot length^33^.

### VOCs emission by healthy and infected tomato roots

**Figure 3:**
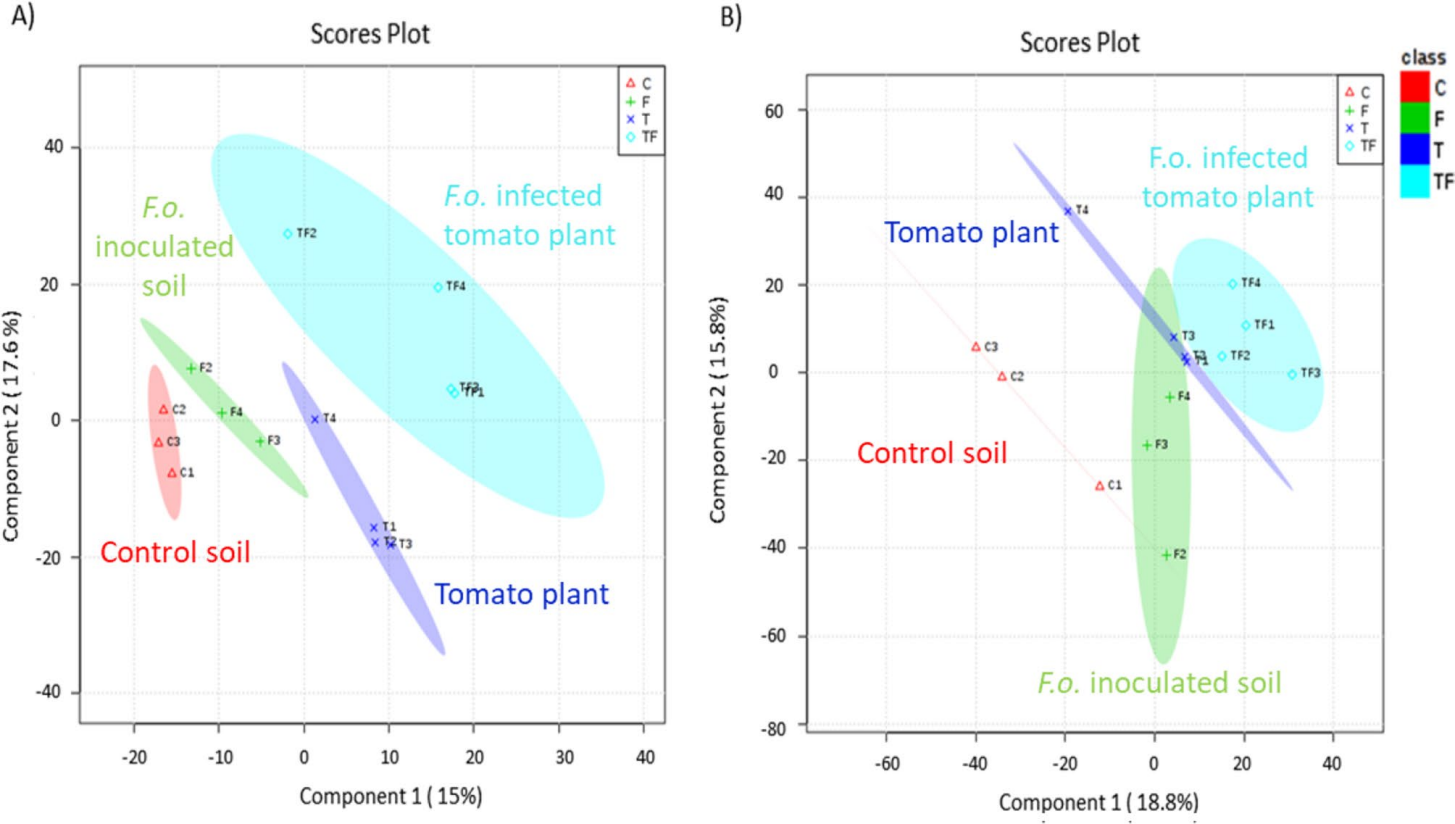
The partial least squares discriminant analysis (PLS-DA) of the VOCs profiles collected from (**A**) the headspace region and (**B**) the root region of the rhizosphere of healthy and with *Fusarium oxysporum* (strain Fol007) inoculated tomato plants (cv. Hildares) and from soil colonized by the pathogen. Gamma sterilized soil (C); *F. oxysporum* inoculated soil (F); healthy tomato plants (T); infected tomato plants (TF).

Plant stress can induce changes in the emission of VOCs that are qualitatively and quantitatively unique. Moreover, different plant organs emit diverse blends of VOCs under stress^34^. In our study, VOCs emitted from healthy and infected tomato roots were collected and analyzed from the headspace and root region (Fig. 1A). Partial least-squares discriminant analysis (PLS-DA) confirmed that the volatile blends differed significantly (posthoc Tukey test, *p* ≤ 0.05) between healthy and pathogen-infected tomato plants, independently of the sampling location (headspace or root region) (Fig. 3). This is in line with other studies where differences in the volatile emissions between healthy and *F. culmorum* infected *Carex arenaria* roots^11^ or between healthy and *Diabrotica virgifera virgifera* affected maize roots^35^ were observed in a soil-based environment. Interestingly, our results also revealed that the volatile profile of a pure culture of *F. oxysporum* in soil significantly differs from the VOCs profile of *F. oxysporum* infected tomato plants, both in the headspace and root region. So far, most analyses of the VOCs emitted by *F. oxysporum* have been performed in vitro or during plant–microbe interactions on artificial media^36^. However, the VOCs produced under in vitro conditions could be quite different from those produced under natural soil conditions^21^.

In the headspace region (Fig. 1A), only 81 VOCs were detected. In contrast, 167 different VOCs were detected near the root zone. Physio-chemical properties of a VOC affect their ability to interact with the soil environment and thus the distance that a VOC can travel. For instance, it has been found that volatile sesquiterpenes have better diffusion capacities in sand than volatiles released by green leaf^37^. The belowground VOCs revealed in the headspace region may play an important role in long-distance chemical communications.

The VOCs produced by tomato roots were annotated and identified using the NIST library database (Supplementary Table S2). VOCs such as cymene, 3-carene, sabinene, myrcene and methyl salicylate have previously been reported to be released by tomato roots^38^. Previous studies on root VOCs were mostly invasive to the root and performed under artificial growing conditions^51^. However, in the present study, the root VOCs were collected in a non-invasive manner and from tomato plants grown in natural soil.

**Figure 4:**
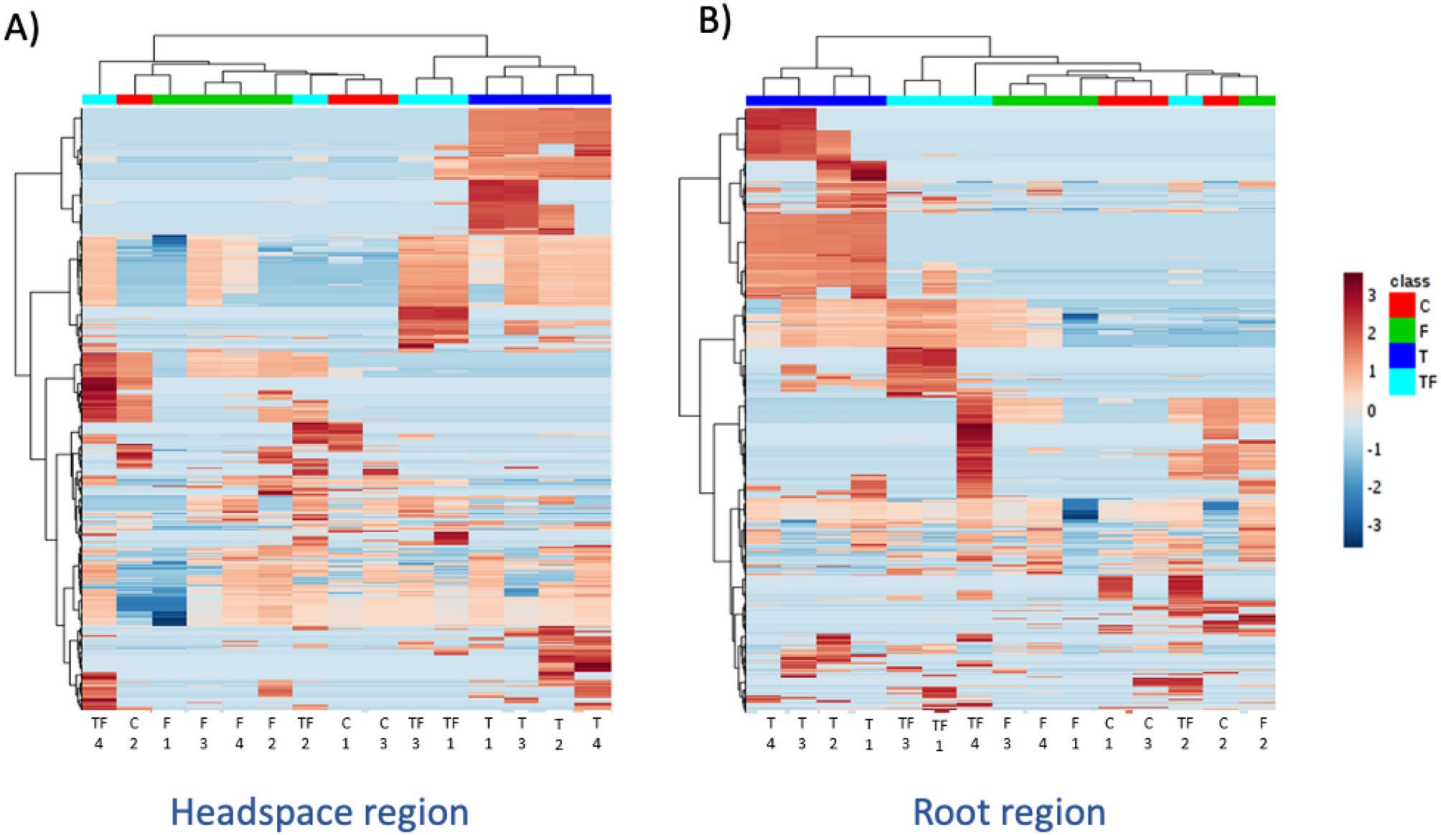
Heatmap of enriched volatiles produced in (**A**) the headspace region and (**B**) the root region of tomato rhizosphere (cv. Hildares). On the vertical axis, different compound are represented. On the upper horizontal axis, different replicates of the treatments are represented forming a dendrogram. The blue to red scale represents the abundance of different compounds, i.e. red color signifies higher abundance of compounds and blue color signifies lower abundance of compounds. Gamma sterilized soil (C); *Fusarium oxysporum* (strain Fol007) inoculated soil (F); healthy tomato plants (T); infected tomato plants (TF).

**Figure 5:**
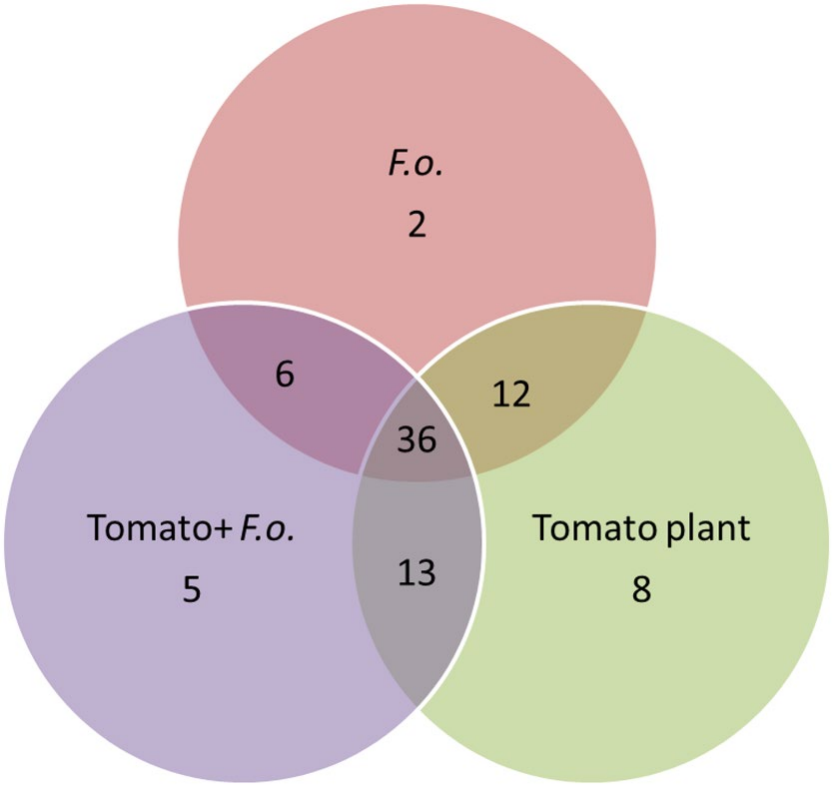
Venn diagram depicting the number of differential and shared VOCs comparing tomato plants (cv. Hildares) without and with *Fusarium oxysporum* (strain Fol007) (+*F.o.*) inoculation and *F. oxysporum* in soil (*F.o.*).

Furthermore, the heatmap analysis revealed clear differences in VOCs profiles between the healthy and infected tomato root (Fig. 4). Interestingly, many compounds produced by the healthy plant roots are not present in the VOC profile emitted by infected plant roots and vice versa (Fig. 5). Additionally, of these compounds, very few VOCs were detected in the soil inoculated with only the *F. oxysporum* strain (Supplementary Table S2). This highlighted that the majority of VOCs found were released by infected roots. But one cannot exclude that the VOC-blend contains VOCs that were produced by the *F. oxysporum* strain.

The VOCs produced by the tomato roots mainly comprised alkanes, esters, ketones and organic acids. Healthy tomato plants produced compounds such as n-alkanes, beclomethasone dipropionate, cymene, decanal and an unknown compound that was not found in any other treatment (Supplementary Table S2). The infected plant roots emitted VOCs such as benzonitrile, benzothiazol, dimethyl trisulfide, formic acid and a terpene-like compound. Most of these compounds are known for their antifungal activities^19^. The pathogen by itself emitted VOCs such as decane, eicosane and napthalene into the soil. However, similar to other studies, many of the VOCs produced by the plant roots or the pathogen were unknown and could not be identified^5,11^.

Interestingly, formic acid was only emitted by tomato roots infected with *F. oxysporum* (Treatment TF). Formic acid is known to be produced by many microorganisms^39,40^ and has previously been reported to have a positive effect on the growth of pathogenic microorganisms, especially fungal pathogens, whereas the opposite was observed in the case of beneficial bacteria, such as *B. pumilus* and *B. megaterium*^41^. In contrast, napthalene, produced by tomato roots in the presence of *F. oxysporum* is known to have antimicrobial function^42^. In turn, this compound may act as a repellent to other microorganisms. The monoterpene, *p*-Cymene, only produced by healthy plants in the study, is known to have an antimicrobial effect. However, when investigated in vitro against *B. cereus,* it did not show any antimicrobial effect^43^, indicating that *p*-Cymene may have antimicrobial activity against specific microorganisms. The volatile 3-Carene, a monoterpene, was detected in all treatments: healthy and infected plants and *F. oxysporum* alone**.** Apart from 3-Carene, a terpene-like compound was also identified in the infected plant. Induction of terpene production in various plants has been previously observed after exposure to a fungal infection^11^. This indicates that terpene compounds might play an important role in plant-pathogen interactions and attraction of beneficial bacteria that are important for plant protection.

Besides emitting VOCs, plants respire carbon dioxide from the roots as a by-product of primary plant metabolism^44^. CO_2_ has been well studied for its role in increasing hyphal growth in some fungi and could be used as a carbon source by many microbes^45,46^. However, recent studies indicate that CO_2_ and plant VOCs may work in synergy as attractants^47^. Although the current study focused only on analyzing the root-emitted VOCs and not CO_2_, we investigated the biological activity of the complete blend of root emitted VOCs (healthy and infected plants) in attracting beneficial bacteria. Hence, an additive or synergistic effect of VOCs and plant-emitted CO_2_ is possible in our system.

### Tomato root metabolite profiling using DART-MS

**Figure 6:**
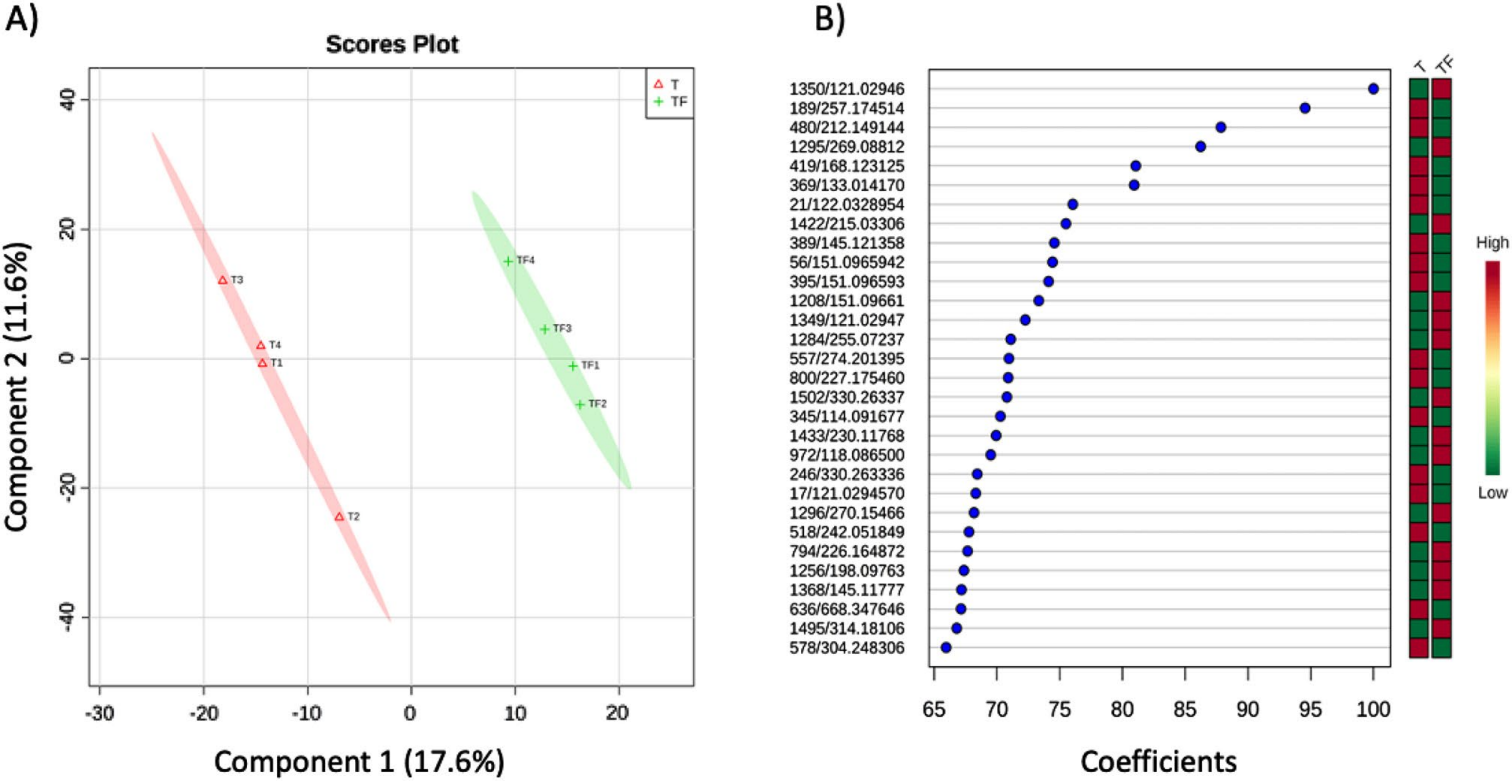
DART-MS of tomato roots (cv. Hildares) (**A**) PLS-DA plot showing the differences in the root metabolite patterns between the healthy (T; red) and infected plant roots (TF; green) (**B**) plot showing significant differences (*p* ≤ 0.05) in the mass features between the healthy (T) and infected plant roots (TF) Red color represents the presence of mass features in low abundance whereas green color represents high abundance.

We applied Direct Analysis in Real Time-High Resolution Mass Spectrometry (DART-HRMS) as a complementary method to the GC–MS analysis of VOCs, to analyze the metabolites from intact tomato roots that could not be covered or detected using GC–MS. We conducted DART-HRMS analysis in both the positive and negative mode, which enabled putative identification of various primary and secondary metabolites in the plant roots. As presented in the PLS-DA plots, the composition of identified metabolites varied between the treatments (Fig. 6). This is in line with the already observed differences in volatile patterns based on GC–MS analysis. DART MS is not the usual method for identifying metabolites in plant materials despite its advantages such as simple sample processing. DART-HRMS allows rapid and direct analysis of volatile and non-volatile metabolites in intact plant material by ionization with a heated gas beam, without the need for sample preparation^48^. To date, this is one of the very few studies that has used DART-MS for analyzing the metabolites of plant roots. Previously, this approach was used for compound detection, species identification and metabolite profiling as well as initial quantifications for herbal medicine analysis^49,50^. We suggest that the DART-HRMS methodology can be used for streamlined analysis of plant metabolites since the sample preparation is minimal and very few parameters need to be modified from experiment to experiment.

### Bacterial attraction by root VOCs in the olfactometer choice assay

**Figure 7:**
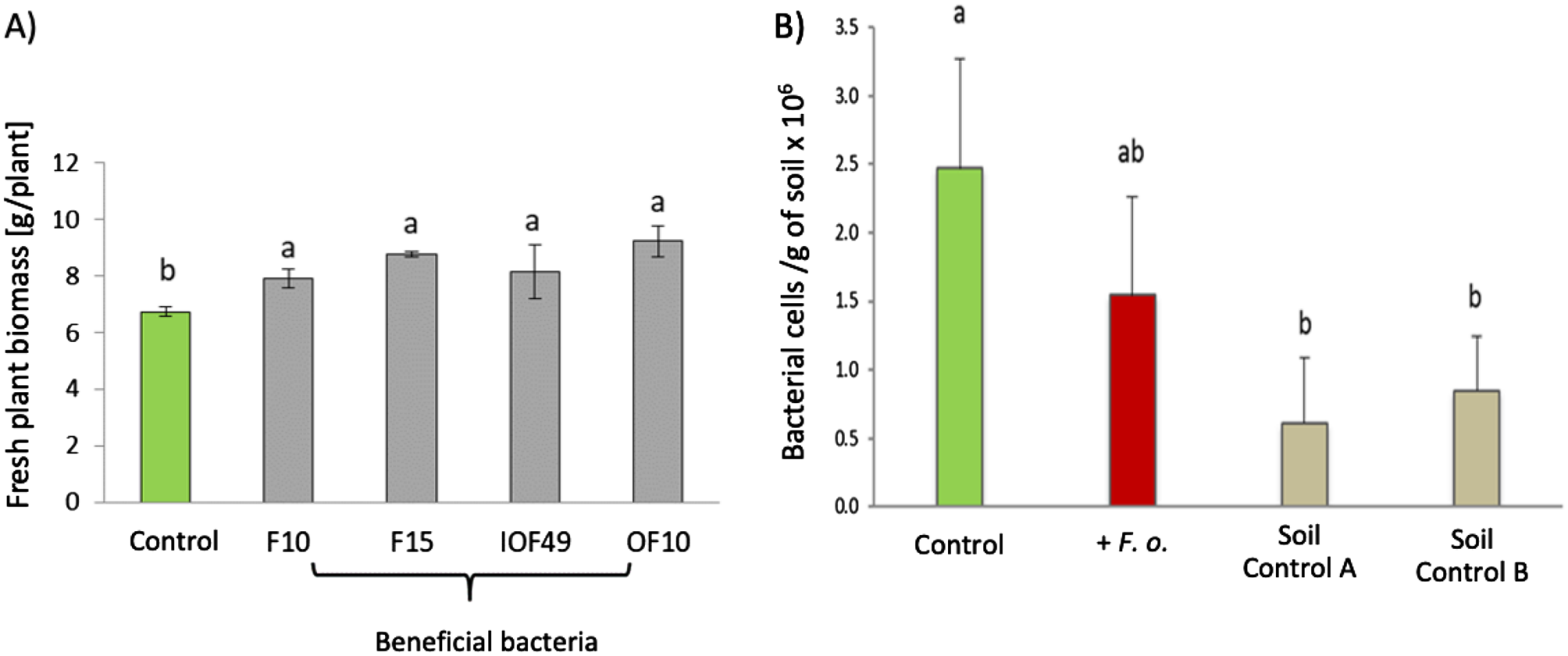
Olfactometer based choice assay (**A**) Effect of beneficial bacteria possessing in vitro antagonism traits against *Fusarium oxysporum* on the overall plant biomass of tomato plants (cv. Hildares); F10: *Bacillus megaterium*; F15: *Bacillus licheniformis*; IOF49: *Bacillus* sp.; OF10: *Bacillus* sp. (**B**) Number of bacterial cells attracted by healthy (Control), infected plants (+*F.o.*) and both ends of olfactometer attached to two soil controls without any plant (Soil Control A and Soil Control B) as assessed by qPCR of the 16S rRNA gene. Different letters on the top of error bars represent significant differences (Tukey test, *p* ≤ 0.05).

An olfactometer-choice assay was set up to test the attraction of applied beneficial bacteria towards plant roots by root-emitted VOCs. The number of bacterial cells was quantified by qPCR at both ends of the tube and compared to the number of bacteria in an olfactometer not connected to plants. Olfactometers have been used extensively to study the interaction of plants with organisms of higher trophic levels^51,52^. However, this kind of system has rarely been used to study plant–microbe interactions cultivated in soil. We used an olfactometer to investigate whether the applied bacteria were attracted by VOCs to the tomato roots (Fig. 1B). The beneficial bacteria used in the consortium showed both plant growth promoting properties for the tomato plant and in vitro antifungal traits against the pathogen *F. oxysporum* (Fig. 7A). Furthermore, all the chosen beneficial strains showed motility under in vitro assays (Supplementary Table S3). Hence, we assume that the infected roots attract the bacteria in higher density to support plant protection.

As already mentioned, the shoot length and root parameters were significantly reduced in infected plants compared to the healthy plants at 7 days post inoculation (3 weeks old plants; 6–10 leaf stage; Supplementary Table S4). The results highlight that bacterial motility was stimulated in the presence of root VOCs compared to soils without any plants (Soil Control A and Soil Control B; Fig. 7B). Regardless, we did not find a significant difference between the number of bacteria that migrated towards the healthy plant roots (2.5 × 10^6^ cells g^−1^ of soil) compared to infected roots (1.5 × 10^6^ cells g^−1^ of soil) (Fig. 7B). This result contradicts our expectation that infected and healthy plant roots differ in their attraction of bacteria with biocontrol properties.

Rejection of the hypothesis that infected roots attract more beneficial bacteria could be because the infected plants produced lower amounts of VOCs than the healthy plants. This could be due to the reduced root biomass and root surface of the infected plant (Supplementary Table S4). Thus the reduced root growth might have further reduced the abundance of volatiles emitted by the infected root^53^. Moreover, the composition of plant VOCs, including different components and their relative ratio, has been observed to exert biological responses in attracting or repelling organisms^54^. Similarly, Knudsen et al.^55^ demonstrated that a flying moth recognizes its plant host based on the ratio between field attractive and background VOCs embedded within a plant odor.

Of the VOCs identified, about 57.31% (47 compounds) were observed to be produced by both healthy and infected plants. The common VOCs included various hydrocarbons (hexane, hexane, pentane, branched alkanes, etc.), benzene and benzene derivatives, as well as organic acids (acetic acid, oleic acid). These VOCs can act as signal molecules that induce migration of bacteria to plant roots. However, it cannot be excluded that the emitted VOCs could be a nutrient source for microbes in this environment. For instance, a study carried out by Schulz-Bohm et al.^11^ tested the attraction of bacteria from a bacterial synthetic community (*Burkholderia*, *Dyella* sp., *Janthinobacterium* sp., *Paenibacillus* sp., *Pseudomonas* sp., and *Collimonas* sp.) towards *F. culmorum* infected and healthy *C. arenaria* roots. It was observed that most bacteria (except *Burkholderia* sp.) under nutrient-deprived conditions showed similar attraction towards both infected and healthy *C. arenaria* roots. In contrast, under nutrient-rich conditions (presence of artificial root exudates), a significant difference in attraction of bacteria was observed towards the infected and healthy *C. arenaria* roots (except *Burkholderia* sp. and *Pseudomonas* sp.). This may be because a pathogen such as *F. oxysporum* can tap into plant derived carbon and reduce photosynthesis by colonizing the root tissue, and therefore, limit the rhizosphere carbon supply^56^. Furthermore, it cannot be excluded that VOCs collected from the infected plants were produced by the pathogen itself, rather than pathogen-induced. Another explanation for the lower amounts of bacteria observed in the rhizosphere of infected tomato plants might be the release of antimicrobial compounds or VOCs by the pathogen *F. oxysporum*. This could have limited the migration of the beneficial bacteria towards the infected plant roots*.* For instance, it has been reported that VOCs produced by fungi impact the motility of bacteria^57^.

We propose that belowground VOCs released by the plant roots can act as both signal molecules and nutrient sources for microbes in the surrounding environment^19,58^.

Overall, our results suggest a differential pattern of root VOCs and metabolites produced by the healthy and infected tomato plant. We started the experiment with the hypothesis that *F. oxysporum* infected tomato plants release inducible VOCs that, in turn, act as info-chemicals to recruit a higher number of beneficial bacteria. However, we observed no significant differences in the migration rate of the applied bacteria between healthy and infected tomato roots. These results were obtained from selected bacteria; therefore, follow-up studies should be performed using total microbial communities to reveal whether the attraction of bacteria is significantly different.

## Conclusion and outlook

This research fills a technical gap to establish an approach for analyzing the role of root VOCs in attracting microbes from the surrounding soil. Most of the research currently available on belowground VOCs was performed using in vitro models that do not mimic natural conditions where the plants grow. We used a soil-based setup to study the root volatile blend produced by healthy and infected tomato plant roots. The established approach offers the opportunity to improve our understanding of the role played by VOCs in belowground plant–microbe interactions. Several of the plant VOCs detected are well known for their antifungal activity. Further studies should address the role of VOCs in the attraction of beneficial microbes or various pathogens using natural microbial soil communities.

## Supplementary information

Supplementary Information.
