# Supplementary material for: A non-invasive soil-based setup to study tomato root volatiles released by healthy and infected roots
Source: Sci Rep. 2020 Jul 29;10:12704. doi: 10.1038/s41598-020-69468-z (PMC7391657; doi:10.1038/s41598-020-69468-z)
Supplement: Supplementary file 1 — Supplementary Information. [file 41598_2020_69468_MOESM1_ESM.docx]

**Title:**

**A non-invasive soil-based setup to study tomato root volatiles released by healthy and infected roots**

**Authors:**

Sneha Gulati^1^, Max-Bernhard Ballhausen^1^, Purva Kulkarni^2^, Rita Grosch^1^, Paolina Garbeva^2^

**Supplementary Information**

**
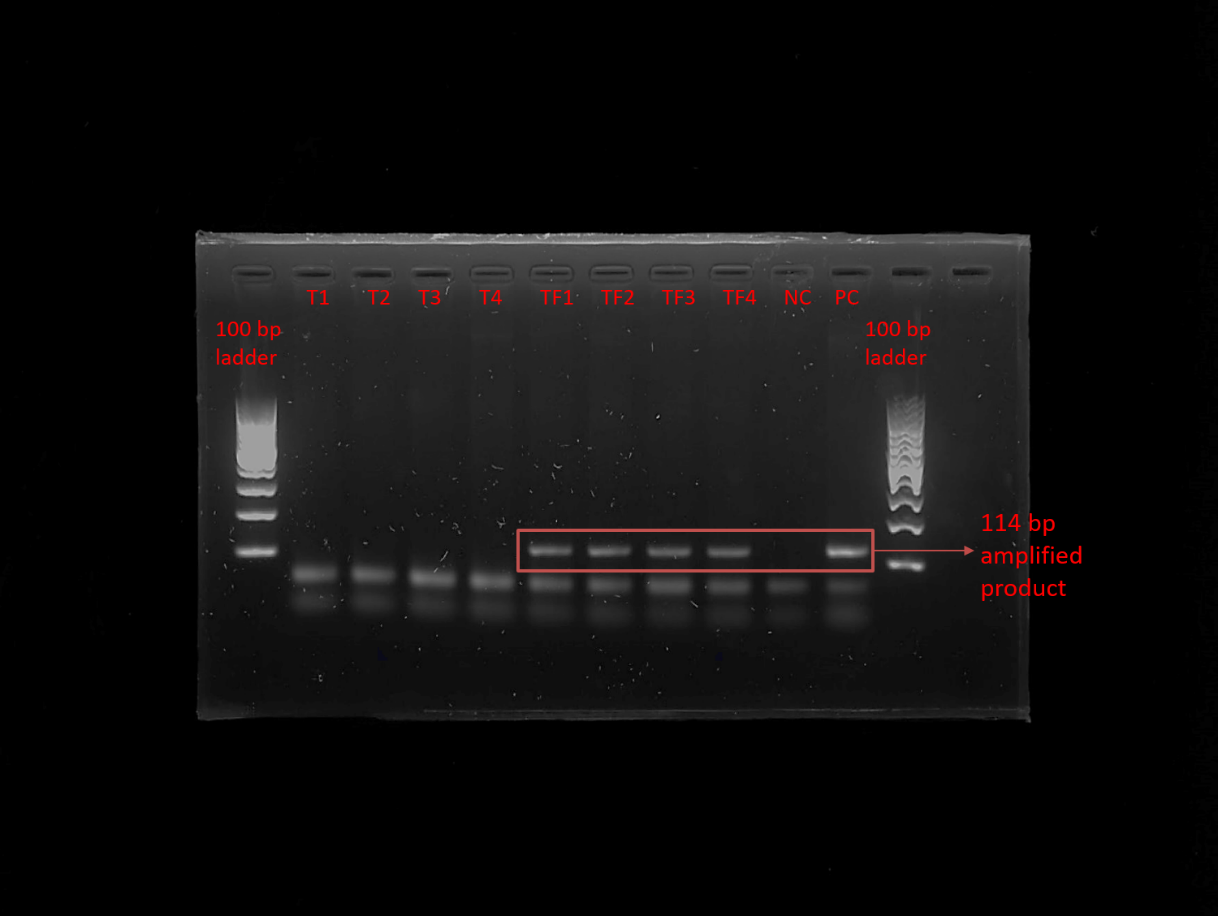
**

**Supplementary Figure S1:** Detection of *Fusarium oxysporum* (strain Fol007) colonization in tomato roots (cv. Hildares) after 1 week of inoculation using the primer pair (FP: GAC GGT GTT TAT TCG GAT GG; RP: AGT TGC GCG ATA TGT GTT TG) specific for SIX1 gene with 114bp amplified product. The Polymerase chain reaction was performed with 2ng total genomic DNA from tomato roots as template. T1-T4 represents the un-inoculated tomato roots; TF1-TF4 represents the *F. oxysporum* inoculated tomato roots; NC is the negative control; PC is the positive control using a pure culture of *F. oxysporum.*

**Supplementary Table S2**: Identified compounds produced by healthy (T) and *Fusarium oxysporum* (strain Fol007) infected tomato plants (cv. Hildares; TF) compared to volatiles released by *F. oxysporum* (F) into the soil and found in pure soil samples (C), annotated using the NIST library. RT denotes the Retention time; ERI denotes the Experimental Retention Index value (average); Superscripts h and r denote the headspace and root region respectively.

| **No.** | **RT** | **ERI** | **VOCs** | **Mean (% of total compounds) ± SE** | | | | | | | |
| --- | --- | --- | --- | --- | --- | --- | --- | --- | --- | --- | --- |
|  |  |  |  | **F^h^** | **F^r^** | **T^h^** | **T^r^** | **TF^h^** | **TF^r^** | **C^h^** | **C^r^** |
| 1 | 2.25 | 568 | Branched alcane | 1.16 ± 0.51 |  |  | 2.05 ± 0.55 |  |  |  | 2.19 ± 0.75 |
| 2 | 2.31 | 575 | Unknown |  |  | 1.50 ± 0.21 | 2.01 ± 0.35 |  |  |  |  |
| 3 | 2.77 | 612.2 | Acetic acid | 2.63 ± 0.37 |  | 1.97 ± 0.27 |  | 1.65 ± 0.35 |  | 5.84 ± 1.02 |  |
| 4 | 2.88 | 620.7 | Pentane | 2.79 ± 0.16 |  | 4.36 ± 0.63 | 4.38 ± 0.94 | 4.42 ± 0.13 | 4.54 ± 0.77 |  |  |
| 5 | 3.07 | 635 | Formic acid |  |  |  |  | 7.19 ± 0.15 |  |  |  |
| 6 | 3.13 | 640.5 | Trichloromethane | 9.52 ± 0.41 | 3.11 ± 0.76 | 7.98 ± 0.63 | 3.56 ± 1.33 |  |  |  |  |
| 7 | 3.19 | 645.2 | Hexene | 9.60 ± 0.39 | 12.66 ± 4.78 | 9.61 ± 0.38 | 10.29 ± 6.32 | 7.19 ± 0.15 |  |  |  |
| 8 | 3.2 | 620.4 | Hexyne | 8.39 ± 0.65 |  | 9.49 ± 0.41 |  | 7.24 ± 0.16 |  |  |  |
| 9 | 3.59 | 676.7 | Benzene |  | 23.41 ± 3.72 | 0.44 ± 0.02 |  | 3.52 ± 0.10 | 0.44 ± 0.02 |  |  |
| 10 | 3.6 | 677 | Hexadien-5-yne | 0.26 ± 0.02 | 4.67 ± 1.53 | 3.18 ± 1.06 | 3.74 ± 2.26 | 3.52 ± 0.10 | 0.47 ± 0.03 | 15.11 ± 0.93 | 6.26 ± 0.84 |
| 11 | 3.99 | 704 | Branched alcane |  |  |  |  |  |  | 1.12 ± 0.42 |  |
| 12 | 4.04 | 705.9 | Unknown | 0.73 ± 0.45 |  |  | 2.30 ± 1.17 | 6.57 ± 0.11 |  |  |  |
| 13 | 5.04 | 747 | Hexanol | 9.44 ± 0.35 | 2.95 ± 1.27 | 6.98 ± 1.98 | 10.83 ± 3.56 | 7.06 ± 0.10 |  |  |  |
| 14 | 5.11 | 746 | Pyridine | 9.31 ± 0.25 |  | 9.61 ± 0.39 | 11.06 ± 3.63 | 7.26 ± 0.14 | 4.43 ± 0.25 | 36.95 ± 3.59 | 43.16 ± 5.06 |
| 15 | 5.16 | 751.7 | Disulfide, dimethyl | 9.35 ± 0.25 | 2.95 ± 1.29 | 9.54 ± 0.33 | 11.06 ± 3.63 | 7.11 ± 0.21 | 4.63 ± 0.22 | 4.18 ± 0.17 |  |
| 16 | 5.39 | 761.2 | Unknown | 8.77 ± 0.49 |  | 0.60 ± 0.05 |  | 6.96 ± 0.19 |  |  |  |
| 17 | 5.75 | 775.9 | Cycloheptatriene | 6.22 ± 2.29 | 2.95 ± 1.30 | 5.34 ± 2.41 | 4.49 ± 2.16 | 0.70 ± 0.06 | 15.68 ± 0.79 |  | 6.42 ± 1.25 |
| 18 | 6.09 | 789.9 | Unknown | 9.51 ± 0.36 |  | 4.91 ± 2.12 | 4.49 ± 3.28 | 7.21 ± 0.12 | 13.54 ± 1.11 |  |  |
| 19 | 6.39 | 801.5 | Unknown | 0.27 ± 0.02 |  |  | 0.47 ± 0.22 | 0.81 ± 0.38 |  |  |  |
| 20 | 6.74 | 811 | Branched alcane |  |  |  |  | 6.93 ± 0.11 |  |  |  |
| 21 | 11.3 | 931.5 | Terpene alike |  |  |  |  | 0.33 ± 0.01 |  |  |  |
| 22 | 11.76 | 942 | Heptene |  | 0.83 ± 0.44 | 0.15 ± 0.02 | 1.84 ± 1.50 | 0.60 ± 0.09 |  |  |  |
| 23 | 11.77 | 942.4 | α-Pinene | 0.09 ± 0.02 | 0.83 ± 0.44 | 0.12 ± 0.01 | 2.13 ± 1.43 | 2.13 ± 1.24 | 3.53 ± 0.32 | 1.03 ± 0.32 | 11.54 ± 3.41 |
| 24 | 12.45 | 958 | Heptane |  |  |  | 0.43 ± 0.23 | 0.01 ± 0.00 |  |  |  |
| 25 | 12.48 | 958.9 | Unknown | 0.31 ± 0.03 | 0.86 ± 0.31 | 0.57 ± 0.17 | 0.43 ± 0.23 | 0.06 ± 0.04 | 2.45 ± 0.47 |  |  |
| 26 | 12.94 | 969.6 | Benzaldehyde | 0.05 ± 0.03 | 0.30 ± 0.04 | 0.55 ± 0.11 | 0.17 ± 0.09 | 0.25 ± 0.02 | 0.04 ± 0.00 |  |  |
| 27 | 13.21 | 976 | Dimethyl trisulfide |  |  |  |  | 0.31 ± 0.11 | 0.05 ± 0.00 |  |  |
| 28 | 13.63 | 985.8 | Benzene derivative |  |  | 0.28 ± 0.11 |  | 0.68 ± 0.06 |  |  |  |
| 29 | 13.66 | 986.5 | Phenol | 0.05 ± 0.03 | 2.32 ± 1.21 | 0.31 ± 0.10 | 4.99 ± 3.66 | 0.73 ± 0.03 | 0.05 ± 0.00 | 1.74 ± 0.24 | 2.89 ± 0.56 |
| 30 | 13.88 | 991.4 | Benzonitrile |  |  |  |  | 0.55 ± 0.14 | 10.61 ± 0.77 |  |  |
| 31 | 14.38 | 1003.1 | Benzofuran | 2.03 ± 0.81 | 5.30 ± 3.54 | 0.59 ± 0.14 |  | 0.09 ± 0.00 |  | 10.91 ± 3.87 | 18.11 ± 6.22 |
| 32 | 14.7 | 1010 | Decane |  | 13.43 ± 5.86 |  |  | 0.12 ± 0.02 |  |  |  |
| 33 | 14.8 | 1013 | Octanal |  |  | 0.19 ± 0.06 | 0.56 ± 0.23 | 0.10 ± 0.01 | 2.24 ± 0.79 |  |  |
| 34 | 15.01 | 1017.8 | 3-carene | 0.11 ± 0.04 | 0.34 ± 0.14 | 0.09 ± 0.02 | 0.85 ± 0.46 | 0.11 ± 0.02 | 2.19 ± 0.82 |  |  |
| 35 | 15.71 | 1034 | Cymene |  |  | 0.07 ± 0.03 | 0.66 ± 0.49 |  |  |  |  |
| 36 | 17.08 | 1066 | Branched alcane |  | 0.39 ± 0.18 |  |  | 0.07 ± 0.03 | 0.13 ± 0.08 |  |  |
| 37 | 17.44 | 1074.4 | Acetophenone |  | 0.39 ± 0.18 | 0.14 ± 0.09 | 0.17 ± 0.09 |  |  |  |  |
| 38 | 17.47 | 1075 | Unknown |  |  |  |  | 0.01 ± 0.00 |  |  |  |
| 39 | 17.76 | 1081.9 | Undecene |  |  |  | 0.17 ± 0.09 | 0.02 ± 0.01 |  |  |  |
| 40 | 18.16 | 1091 | Unknown |  |  | 0.02 ± 0.00 |  |  |  |  |  |
| 41 | 19.19 | 1108.3 | Nonanal |  |  | 0.79 ± 0.23 |  | 0.01 ± 0.00 |  |  |  |
| 42 | 19.28 | 1118 | Hexanoic acid | 0.38 ± 0.10 | 0.58 ± 0.26 | 0.01 ± 0.00 | 0.77 ± 0.46 |  |  |  |  |
| 43 | 23.08 | 1212 | Dodecane | 0.33 ± 0.03 | 0.24 ± 0.16 |  |  |  |  |  |  |
| 44 | 23.1 | 1213 | Branched alcane |  |  |  | 0.06 ± 0.02 | 0.09 ± 0.00 |  |  |  |
| 45 | 23.29 | 1218.3 | Decanal |  |  | 0.17 ± 0.10 | 0.06 ± 0.02 |  |  |  |  |
| 46 | 23.31 | 1218.8 | Decenol | 0.29 ± 0.04 |  |  | 0.06 ± 0.02 | 0.09 ± 0.00 |  |  |  |
| 47 | 23.97 | 1236 | Benzothiazole |  |  |  | 0.08 ± 0.02 | 0.10 ± 0.01 |  |  |  |
| 48 | 24.86 | 1260.6 | Unknown |  |  |  |  | 0.05 ± 0.02 | 0.13 ± 0.08 |  |  |
| 49 | 25.81 | 1287 | Branched alcane | 0.05 ± 0.03 | 0.03 ± 0.01 | 0.04 ± 0.03 |  | 0.07 ± 0.02 | 0.13 ± 0.08 |  |  |
| 50 | 27.52 | 1337.7 | Branched alcane |  |  | 0.20 ± 0.06 |  |  |  |  |  |
| 51 | 27.75 | 1344 | Butyl nitrite | 0.08 ± 0.03 | 0.40 ± 0.15 |  | 0.20 ± 0.09 | 0.17 ± 0.02 | 0.13 ± 0.08 |  |  |
| 52 | 32.99 | 1508 | Branched alcane |  | 0.02 ± 0.00 |  |  | 0.03 ± 0.02 |  |  |  |
| 53 | 36.3 | 1619 | C16 |  |  |  |  | 0.01 ± 0.00 |  |  |  |
| 54 | 38.28 | 1784 | Hydrobenzyl alcohol | 0.43 ± 0.10 | 0.35 ± 0.09 | 2.22 ± 0.59 | 1.13 ± 0.55 | 0.15 ± 0.06 | 0.64 ± 0.12 | 8.37 ± 5.33 | 4.19 ± 0.77 |
| 55 | 38.84 | 1897 | Eudesma |  |  |  | 1.01 ± 0.56 |  | 0.59 ± 0.08 |  |  |
| 56 | 39.48 | 1942 | Salicylic acid |  |  | 0.31 ± 0.10 | 0.17 ± 0.09 |  |  |  |  |
| 57 | 40 | 1977 | Hexadecanoic acid | 0.02 ± 0.00 |  |  | 0.06 ± 0.02 |  |  |  |  |
| 58 | 40.24 | 1993 | Heptadecanol | 0.02 ± 0.01 |  |  | 0.06 ± 0.02 |  |  |  |  |
| 59 | 40.34 | 2000 | Eicosane |  | 0.95 ± 0.38 |  |  |  | 0.35 ± 0.05 |  |  |
| 60 | 40.39 | 2039 | Isopropyl palmitate | 0.02 ± 0.00 |  | 0.26 ± 0.08 | 0.05 ± 0.02 | 0.03 ± 0.02 |  |  |  |
| 61 | 40.9 | 2023 | Pentadecanoic acid |  |  |  |  | 0.04 ± 0.02 |  |  |  |
| 62 | 42.02 | 2109 | Branched alcane |  | 0.23 ± 0.08 | 0.31 ± 0.08 | 0.89 ± 0.42 | 0.04 ± 0.02 | 1.47 ± 0.22 |  |  |
| 63 | 42.02 | 2109.7 | C21 |  |  | 0.34 ± 0.07 |  | 0.04 ± 0.02 |  |  |  |
| 64 | 42.69 | 2144.5 | n-alcane |  |  | 0.46 ± 0.10 | 0.89 ± 0.42 |  |  |  |  |
| 65 | 43.3 | 2193 | Branched alcane |  |  |  | 1.12 ± 0.36 |  | 1.79 ± 0.73 |  |  |
| 66 | 43.31 | 2200 | Docosane | 0.36 ± 0.05 |  | 0.96 ± 0.22 |  | 0.42 ± 0.20 | 3.61 ± 0.39 |  |  |
| 67 | 43.31 | 2177 | Naphthalene | 0.40 ± 0.07 |  |  |  | 0.42 ± 0.20 |  |  |  |
| 68 | 43.5 | 2189.4 | Beclomethasone dipropionate | 0.33 ± 0.03 |  |  |  | 0.43 ± 0.20 |  |  |  |
| 69 | 43.54 | 2189 | Heneicosane | 0.32 ± 0.05 | 2.03 ± 1.03 |  | 1.15 ± 0.36 | 0.43 ± 0.10 | 3.72 ± 0.29 |  |  |
| 70 | 43.64 | 2193.9 | Dodecahydrophenanthrene | 0.40 ± 0.07 |  | 1.84 ± 1.09 |  | 0.79 ± 0.04 |  |  |  |
| 71 | 43.71 | 2197.7 | Octadecadiynoic acid | 0.39 ± 0.09 |  | 3.58 ± 1.12 | 1.12 ± 0.36 | 0.94 ± 0.07 |  | 4.11 ± 0.93 | 5.25 ± 1.69 |
| 72 | 43.71 | 2197.6 | Kaurene |  |  |  |  |  |  |  |  |
| 73 | 43.93 | 2240.6 | Branched alcanes |  |  |  | 1.12 ± 0.36 |  |  |  |  |
| 74 | 43.95 | 2241 | Octadecane |  | 2.03 ± 1.03 |  | 1.12 ± 0.36 | 0.92 ± 0.02 |  |  |  |
| 75 | 44.63 | 2388.6 | Tetracosane | 0.51 ± 0.02 |  | 1.80 ± 0.75 | 1.18 ± 0.35 | 0.94 ± 0.03 | 3.84 ± 0.37 |  |  |
| 76 | 44.63 | 2389 | C24 |  |  | 1.83 ± 0.74 | 1.18 ± 0.35 | 0.94 ± 0.03 |  |  |  |
| 77 | 44.91 | 2446 | Phthalic acid | 0.53 ± 0.04 | 0.84 ± 0.09 |  | 1.18 ± 0.35 |  | 3.84 ± 0.37 |  |  |
| 78 | 46.36 | 2758 | Heptacosane | 0.85 ± 0.10 | 3.42 ± 2.16 | 2.12 ± 1.31 | 2.23 ± 0.76 | 0.59 ± 0.17 | 3.52 ± 0.37 | 10.63 ± 5.07 |  |
| 79 | 47.48 | 2999.8 | Triacontane | 0.58 ± 0.03 | 0.78 ± 0.16 |  |  | 0.31 ± 0.05 | 2.09 ± 0.05 |  |  |
| 80 | 47.69 | 3041 | Tetracosahexaenol | 0.51 ± 0.01 | 0.74 ± 0.10 |  |  | 0.20 ± 0.06 | 1.91 ± 0.16 |  |  |
| 81 | 50.76 | 3695 | Oleic acid | 0.28 ± 0.02 | 0.86 ± 0.03 | 1.41 ± 0.40 |  | 0.28 ± 0.02 | 1.78 ± 0.27 |  |  |
| 82 | 53.9 | 4372 | Tetratetracontane | 2.32 ± 0.09 | 4.18 ± 0.70 | 2.78 ± 0.39 |  |  | 5.43 ± 0.77 |  |  |

**Supplementary Table S3:** Motility of bacterial strains used in the study as observed *in vitro* on 10% Nutrient agar medium supplemented with different concentrations of agar (0.3%, 0.5%, 1.0%) at 72 hours of incubation at 25 °C.

| Bacterial strain | Replicate | Swimming  (0.3% agar)  (cm) | Swarming  (0.5% agar)  (cm) | Twitching  (1% agar)  (cm) |
| --- | --- | --- | --- | --- |
| *Bacillus megaterium* F10 | 1 | 4.5 | 1.5 | 0.6 |
|  | 2 | 5.1 | 3.5 | 0.8 |
|  | 3 | 5.0 | 3.2 | 0.6 |
| *Bacillus licheniformis* F15 | 1 | 4.1 | 4.0 | 0.9 |
|  | 2 | 3.5 | 4.1 | 0.7 |
|  | 3 | 3.3 | 3 | 0.6 |
| *Bacillus* sp. IOF49 | 1 | 4.2 | 2.5 | 0.9 |
|  | 2 | 3.3 | 2.0 | 1.0 |
|  | 3 | 4.1 | 2.2 | 0.8 |
| *Bacillus* sp. OF10 | 1 | 4.2 | 4.0 | 1.5 |
|  | 2 | 4.5 | 4.0 | 1.0 |
|  | 3 | 4.9 | 4.5 | 1.5 |

**Supplementary Table S4**: Effect of *Fusarium oxysporum* (+*F.o.*) on root parameters of tomato plants (cv. Hildares) used in the olfactometer assembly using the root scanner WinRHIZO.

| Treatment | Replicates | Total Projected Area  (cm^2^) | Total Length  (cm) | Total Surface Area  (cm^2^) | Average Diameter  (mm) | Root Volume  (cm^3^) |
| --- | --- | --- | --- | --- | --- | --- |
| Tomato plant | 4 | 168.45 a | 64.95 a | 9.47 a | 0.46 a | 0.11 a |
| Tomato plant + *F.o.* | 4 | 111.84 b | 34.24 b | 4.64 b | 0.43 a | 0.05 b |

Within each column values followed by different letters are significantly different. (One-way ANOVA; Tukey test, *p* ≤ 0.05)
